# A father nevertheless: Self-confident but resigned fathers with children in foster care

**DOI:** 10.1177/17446295231225525

**Published:** 2023-12-29

**Authors:** Päivi Adolfsson, Helena Lindstedt, Gunnel Janeslätt, Karin Jöreskog

**Affiliations:** Department of Public Health and Caring Sciences, Health equity and working life, Uppsala University, Sweden; Department of Public Health and Caring Sciences, 8097 Uppsala University, Sweden; Department of Public Health and Caring Sciences, 8097 Uppsala University, Sweden; Center for Clinical Research, Region Dalarna, Sweden; SUF Resource Centre, Region Uppsala, Sweden; SUF Resource Centre, Region Uppsala, Sweden; Department of Public Health and Caring Sciences, Public health, working life and rehabilitation, 8097 Uppsala University, Sweden

**Keywords:** child protection, cognitive limitations, parents, qualitative study, in placement

## Abstract

This qualitative study aimed to explore the experiences of nine fathers with neurodevelopmental disabilities with children in foster care, including their fathering role, visits and formal and informal support. Systematic text condensation was the analysis method used. The respondents’ experience of fatherhood revealed two categories: I accept my situation and I am frustrated. Though self-confident, the fathers expressed ambivalence between acceptance and frustration with their role. The study shows that more should be done to provide adapted support for these vulnerable fathers with children in foster care, although they seldom demand such support. Social workers and professionals from the rehabilitation team within the healthcare service should be aware of gendered settings, specifically norms of masculinity. Increased efforts from the social workers may reduce the risks of detachment in parenthood because engaged and informed fathers are in the children’s best interests.

## Introduction

Placing a child in foster care may be necessary for child protection reasons but this is a significant intervention with enormous consequences for the child (Haight et al., 2003) and family (Mayes and Llewellyn, 2009), with many parents reporting intense feelings of loss (Baum and Burns, 2007). To diminish the negative consequences of placement in foster care for the child and parents’ visits, scheduled face-to-face contact is offered during placement (Haigh et al., 2003).

Little is known about fathers with neurodevelopmental disabilities whose children are placed in foster care facilities. According to DSM-5, neurodevelopmental disabilities begin in childhood, with intellectual difficulties and difficulties in conceptual, social and practical living areas (DSM 5, 2013). Neurodevelopmental disabilities include intellectual disability, attention deficit hyperactivity disorder, autism, specific learning disorders and other neurodevelopmental disorders. Neurodevelopmental disabilities often include cognitive limitations. Obtaining information from public records is difficult; however, Tøssebro et al. (2017) found that at least 25% of child protection cases in Norway involve parents with intellectual disability. These records show that it is more usual that women with intellectual disability become mothers than men fathers (Ibid.). It is therefore reasonable to assume that fathers with neurodevelopmental disabilities are also included in some of the research on a more general group of fathers with children in foster care. Persons with neurodevelopmental disabilities experience learning, remembering and problem-solving difficulties. Identifying these fathers and collecting their experiences are essential to gaining new knowledge about tailoring support.

Parents with intellectual disability face a greater risk of losing parental custody of their children than other parent groups (Mayes and Llewellyn, 2012). Research shows that child protection services identify children of parents with intellectual disability at higher risk of maltreatment than children whose parents do not have intellectual disability, which may be why they are willing to help parents of these children more than other parents (Proctor and Azar, 2013). Measures of intelligence quotient undermine the strengths of parents with intellectual disability and structural issues (e.g., poverty and intellectual and developmental status) are yardsticks for identifying the unfit parent) (Sigurjónsdóttir and Rice, 2017). Pacheco et al. (2021) showed that if child protection services identified parents with neurodevelopmental disabilities, child protection authorities are less likely to consider alternatives to child removal or investing in the family. These parents typically perceive the child protection process as unjust (Llewellyn et al., 2010). In addition, they are more likely to be referred to social services than parents without disabilities (DeZelar and Lightfoot, 2019).

Symptoms of attention deficit hyperactivity disorder may lead to parental strain, distress and diminished well-being. Kroeger (2018), examining young adult parents with symptoms of attention deficit hyperactivity disorder, found that they feel not as close to their children and experience less happiness than parents not diagnosed with attention deficit hyperactivity disorder. Parents with attention deficit hyperactivity disorder or autism often have problems with cognitive functions (e.g., attention, working memory, planning) and self-regulation. They also struggle with parenting and child rearing (Johnston et al., 2012). A review linked parental attention deficit hyperactivity disorder to impairments in parenting (Friedrich et al., 2017). The review showed that parental attention deficit hyperactivity disorder was negatively associated with consistent discipline, parental involvement and positive parenting. In addition, parental symptoms were positively associated with lax and over-reactive parenting, often in an unpredictable mix (Ibid.).

In family-focused research there has been a shift from emphasis on mothers to gender-blind attention on parenting, which often fails to be explicit about the experiences and roles of mothers and fathers. In contrast, disability in fathers has received little attention (Kilkey and Clarke, 2010).

Mothers with neurodevelopmental disabilities and children in foster care experience a destabilizing threat to their mother's identity when challenged by the prejudice of others who suggest that they should not bear children (Gould and Dodd, 2014). They also reported maternal feelings of emptiness, sadness and powerlessness (Mayes and Llewellyn, 2012). Although mothers no longer had daily custody of their children, they often remained immersed in their relationship with them and their maternal role. Janeslätt et al. (2019) found that mothers with neurodevelopmental disabilities whose children were in foster care experienced a threat to their identity and a need to alter their maternal role to adjust to life without their children. The authors also found that adapted support was needed to understand the decisions made by child protection services and to facilitate cooperation with professional services and the foster home. In Sweden, children in foster care have the moral and legal right to a continued relationship with their birth parents. Against this background, social services must support parent-child visits (Act, Swedish Social Services, 2001:453).

In a review of parents with attention deficit hyperactivity disorder (Friedrich et al., 2017), only 6 of 14 studies had both mothers and fathers as study participants. The remaining eight studies investigated only mothers. The review showed that fathers’ attention deficit hyperactivity disorder led to impairments in parenting but inconsistent gender differences compared to mothers. However, both parents could influence each other’s parenting style (Ibid.). Attention deficit hyperactivity disorder in mothers and fathers may also be related to negative parenting and child-rearing conflicts. Williamson et al. (2017) demonstrated that mothers with persistent inattention patterns were linked to problems only when fathers also expressed inattentive characteristics.

More and Tarlenton (2022) interviewed 10 parents (6 mothers, 4 fathers), some living with their children while others had children in foster care. Parental self-understanding was linked to gendered parenting roles. While mothers’ self-understanding centered on being primarily responsible for the child, fathers often felt excluded from their child’s life, emphasizing the gendered role of the financial provider (Ibid.). Franklin et al. (2022) reported that while parents with intellectual disability (17 mothers, 5 fathers) experience stigma and disempowerment, they still embrace their parental identity.

There is limited knowledge about the experiences of fathers with neurodevelopmental disabilities who have children in foster care. In general, fathers with children in foster care are rarely involved in child welfare services, and social workers seldom engage with the fathers’ lack of participation (Nygren et al., 2019). In one comparative study the participation and absence of fathers in social work practice in England, Ireland, Norway and Sweden were explored using focus group interviews with social workers (Ibid.). Despite policy documents emphasizing the need to involve fathers in child welfare services in all four countries, the role of fathers continues to be marginalized in real-world social work practice (Ibid.). The authors identified two dominant themes contributing to the exclusion of fathers: one parent (i.e., the mother) being primarily responsible for the child’s care and the father being perceived as a risk.

Symonds et al. (2021) explored the perspective of fathers with neurodevelopmental disabilities with and without children living at home. They found that fathers with neurodevelopmental disabilities do not always receive proper support for their parenting and were not always included in family social services. The authors stated that when social services engaged these fathers in support directed to their families, they could improve their fatherhood and the father-child bond (Ibid.).

The social workers’ perspective on fathers’ involvement in child welfare services has been described by O’Donnell et al. (2005). The social workers agreed on the lack of paternal participation but disagreed on whether they, as welfare professionals, should address this issue and, if so, what concrete steps to take. Fathers’ engagement in child protection services was summarized in a systematic review describing the challenges fathers have historically faced (Gordon et al., 2012). The review outlined factors that promote fathers’ engagement on different levels: individual, family, service providers and community and policy levels (Ibid.). The study showed the need to adjust services to culturally and linguistically diverse families and families with different income and education levels. Regrettably, the need to modify the services offered to fathers with neurodevelopmental disabilities was not covered (Ibid.).

The results from in-depth interviews with 15 fathers, all but two without disabilities living with their children when the court ordered the removal of a child, showed that all 15 fathers experienced the court decision as a traumatic event associated with intense pain and loss (Baum and Negbi, 2013). They grieved the loss of their children’s day-to-day presence and the loss of their role as fathers. However, the fathers’ grief seemed to follow a process toward adaption to the removal (Ibid.). Pytlowana and Kroese (2021) found the court procedures unfair and stressful for parents with intellectual disability. Ćwirynkało and Parchomiuk (2022) noted that fathers with intellectual disability (n=20) feel disempowerment and need support to develop their fathering role through educational activities and increasing social and professional competencies developed through social workers. The authors recognized the importance of reciprocal cooperation between the fathers and social workers.

The literature identifies factors contributing to the vulnerability of fathers with neurodevelopmental disabilities who have children in foster care. There is some research on fathers in general with children in foster care. However, research on fathers with neurodevelopmental disabilities with children in foster care is lacking. With regard to parents with neurodevelopmental, it has been shown that social workers often have excluded fathers from the child’s life. Mothers with neurodevelopmental disabilities have been seen as the only responsible parent, whilst fathers have been considered to be a potential risk. The mother’s grief about the child’s foster care is present in the research, but the fathers’ experiences are unknown. Therefore, it is essential to identify the experiences of fathers with neurodevelopmental disabilities who have children in foster care.

## Aim

This study aimed to explore the experiences of fathers with neurodevelopmental disabilities who have children in foster care, including their experiences of the fathering role, parent-child visits and formal and informal support.

## Method

A qualitative study design was used, and the analysis method was systematic text condensation (Malterud, 2012). The study design was approved by the Regional Ethical Review Board in Uppsala (Reg. no 2014/369).

In the present study birth fathers with neurodevelopmental disabilities and children placed in foster care are referred to as “fathers”. Birth parents are called “parents” to distinguish them from foster parents.

### Respondents

In this study persons with neurodevelopmental disabilities include those with mild intellectual disability, autism, attention deficit hyperactivity disorder and learning disabilities (dyslexia). These individuals often have additional diagnoses, including depression or bipolar disease. A purposive sample was used to recruit respondents (Patton, 2015). The inclusion criteria were fathers with neurodevelopmental disabilities and experiences of having one or more children in foster care. Both biological fathers and legal caregivers, in the present or the past, were included. The fathers were recruited in the middle part of Sweden via social workers and staff from the rehabilitation team within the healthcare service. A few respondents were recruited through mothers who had participated in a support group called “After all, I’m a mother” (Adolfsson et al., 2021). Both professionals and mothers used an easy-to-read leaflet (i.e., text presented in an accessible and easy-to-understand format) to recruit fathers with neurodevelopmental disabilities experiencing cognitive limitations who had children in foster care. In all, 13 fathers were invited to the study (four declined participation). With informed consent, nine fathers (Table 1) with 20 children participated in the study.

**Table 1.** The characteristics of the nine respondents.

Variables		(n)
Age	M= 43, SD= 9,6, range= 24-54	
Number of children	Age range=1-27 years	20
	In placement	15
	Living at home	5
Respondents level of education		
	Mainstream school	7
	Special school	2
Diagnoses, reported by respondents		
	Mild intellectual disability	2
	Attention deficit hyperactivity disorder /Attention deficit disorder	3
	Dyslexia	2
	Minor brain damage	2
	Bipolar disease	1
Living conditions		
Housing	Apartment	8
	House	1
Civil state	Single	3
	Living with a partner	6
Employment status		
	Employment, full time or part time	5
	Daily occupation: (caretaker, assistant) in differing range	3
	No daily occupation	1

### Data collection

An important ethical issue is associated with interviewing fathers with neurodevelopmental disabilities and children in foster care. Because persons with neurodevelopmental disabilities, including intellectual disability, are a vulnerable social group, the current researchers paid particular attention to the respondents’ welfare before, during and after the interviews. An interview guide to assess the maternal role of mothers with neurodevelopmental disabilities who have children in foster care (Janeslätt et al., 2019) was used. The interview guide contained five overarching questions with eight follow-up questions. It was adapted to accommodate persons with cognitive limitations, including simplified language, open-ended questions and few response alternatives (Azar et al., 2013). The fathers were first asked demographic questions: age, housing, number of children, how many children were or had been in foster care, education level, employment and type of disability. Semi-structured questions then covered fathers’ experiences of their fathering role, parental visits and whether they had any support needs in their role as a father. Examples of questions included: How do you experience your parenting role today? Can you decide what you do during the visits? Can you influence the child’s everyday life in the foster home?

Depending on the father’s choice, the interview was held at a rehabilitation center, at the father’s workplace, in the father’s home or by telephone. Before the interviews, fathers received reminders about the appointment. The interviewers were responsible for the tranquility and privacy of the environment during the interviews. The interviews were conducted by the first, second and fourth authors and by two other professionals with experience interviewing persons with neurodevelopmental disabilities. However, no one was professionally connected to the respondents. One of the fathers had a family member present during the interview. The fathers received information about the purpose of the study both orally and in writing before the start of the interview. The fathers were also told that the interview would be audio-recorded for later transcription. During the interviews, the fathers received help in staying focused on the subject, and questions were rephrased or exemplified to be more easily understood when needed. The interviews lasted between 35 to 65 minutes. After the interviews, the respondents were offered a gift card. The interviews were conducted between February 2015 and March 2017 as a part of a large project that included parents with neurodevelopmental disabilities and cognitive limitations. The present study is the last in this project.

### Data analysis

All interviews were transcribed verbatim. As Malterud (2012) described in detail, systematic text condensation was used. A descriptive method was chosen because a limited number (n=9) of respondents were included. This method, which consists of four steps, is suitable when obtaining a whole picture of a phenomenon is impossible. In the first step the interviews were read and reread in an open and naïve manner to identify preliminary themes. Examples of the preliminary themes about the fathers’ experiences and feelings that emerged from the text were 1) unpretentiousness, doubtfulness, 2) feeling despair and dissatisfaction, 3) feeling trust, 4) wanting the support to be better. 5) the fathers take responsibility and 6) fathers have their own needs. In the second step the material was reviewed to identify units of meaning relevant to the study’s aim. The units of meaning were then shortened to codes within code groups that served as the preliminary themes (Table 2). The first and third authors carried out the first and second steps. The third step involved systematic text abstraction of units of meaning within the code groups, which was discussed repeatedly and jointly by all four authors. The authors agreed to form final categories and subcategories from the preliminary themes according to the code groups. In the final step the first and the third authors formulated a preliminary analytic text that was discussed and edited by all four authors until consensus was reached. The findings include authentic interview quotations to illustrate and confirm the findings.

**Table 2.** Presentation of the working process from preliminary themes, codes to condensed code groups that were converted to categories.

Preliminary themes		Examples of codes	Condensed code group/Category
The fathers take responsibility		To be a father Good conditions for family life	I accept my situation
Feeling despair and dissatisfied		Fell between the chairs Requires skills help	I am frustrated

## Findings

The present authors included some of the interviewers’ comments in the citations to clarify the message because some of the fathers’ remarks contained very few words. In the quotations, the capital letter I represents the interviewer and the other capital letters in the beginning of phrases represent the interviewees.

**Figure 1.**
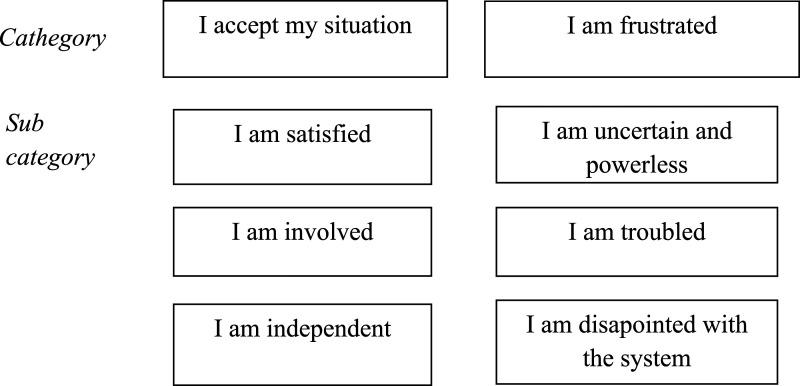
The fathers experienced their fatherhood in two contradictive categories and six subcategories.

The Findings include two categories and six subcategories (Figure 1).

### Category: *I accept my situation*

In this category the fathers expressed their satisfaction with their overall situation. They felt trust and confidence and appreciated the child’s present situation and the well-functioning cooperation between the foster home, the child protection authorities and the service given to themselves and the family. Some fathers reported having closer contact with the child now that they were in foster care. They stressed the importance of feeling independent and having a job, describing managing employment as the most important demand placed on fathers.

### I am satisfied

The subcategory *I am satisfied* reveals that the fathers were comfortable with their life situation. They also stressed the opportunities the visits gave them to see their children. Their contact with the foster families was good. The fathers also felt the foster families facilitated the visits, respected them and welcomed them to take part in family life during the visits.

T: But they said right from the start that we shouldn’t be afraid to tell them if there’s anything we wonder about.

I: No. Has there been any occasion when you felt that you should ask them to do …

T: No, not actually, No. That’s, it’s all been taken care of so well so we haven’t had to do anything.

I: No.

T: It hasn’t felt like that. (Father no. 1)

The fathers reported that the social authorities and social workers understood their situation before and after the children were removed from the home. The support they were offered in their fathering role was satisfying in both cases.

The fathers’ satisfaction depended not only on their situation but also on the children. They could see that their children’s needs were better met in the foster home. In their words there is a focus on the needs of the child.

I: When they, when she was taken into care, did you agree that she needed to be placed in a foster home, or how was it?

M: Yes, it was really too much for her. First at home, then daycare, then nightcare and then school of course, and then the contact family and so on. It got to be too much, it got too messy. (Father no. 2)

The fathers also reported satisfaction with the foster care decision and agreed with the social workers.

### I am involved

In this subcategory the fathers experienced being able to participate as fathers, i.e., they felt they were a part of parenthood. Moreover, they shared a mutual understanding with the foster families concerning the approach to child-rearing. The foster families had time to listen to them, and the fathers said they could talk about anything. One of the fathers stated:

I: Erm, do you feel that you can influence the visitations with your children? Influence…

T: Yes, that depends, how do you mean?

I: Well, for example, can you choose when and where and for how long you can meet?

T: Yes, well yes I can.

I: So, you can be in on deciding that?

T: Yes. (Father no. 1)

Another father stated:

M: No, but I have to say I feel that things actually work well.

I: But you, yourself, do you feel like you are a parent?

M: Yes, well, yeah. I am part of it, I can tell them what I think and we get along really well, so. Or yes, well yeah we do. I tell them that I want to meet him now and then it sort of like, yeah. (Father no. 3)

The opportunity to participate entails that the fathers felt they were trusted and could influence the visits. The fathers reported feeling content with the foster families. Even the fathers who had only had occasional contact with the foster family were convincingly satisfied.

I: Can you influence the visits so that, can you say how you would like things, so to speak?

M: Yes, well I think I can actually. Because we have a really good contact. I think we have it good, it kind of works well. (Father no. 2)

### I am independent

In this subcategory the fathers emphasized their increasing autonomy from the child protection authorities and in life in general. On the one hand, they experienced not being offered support. They had to find solutions in such situations, including support when needed. On the other hand, they felt their competence was good enough to care for their children and themselves. Such competence included the ability to decide what to do during visits.

I: Can you influence what you can or will do when you have time with your child? Do you decide what you do or how does it work?

M: Yes well, he (my son) has ideas for all sorts of things to do and so on. So we don't ask the family home if that's OK or not. All we talk about or what I tell them is what time I want them to pick him up or when they will be eating. Then what we do, that's me that takes care of, like. Then we tell them for example that we've done this or that, so they don't go and do the same things straight after. No, so yes I do think I'm part of it. (Father no. 3)

### Category: I am frustrated

This category reveals that there are contradictions in the fathers’ lives. They felt uncertain and powerless when confronted with the expectations placed on them by the professionals. Still, they did not object but admitted to having ambitions they could not achieve. They were aware of their shortcomings due to their cognitive limitations and experienced a lack of personal support. They were disappointed with the system, the professionals and the authorities. They reported a lack of continuity in the decisions made by the child protection authorities. According to the fathers, the professionals were incompetent. Realizing they could not influence the authorities, they calmly accepted their situation and focused entirely on their employment.

### I am uncertain and powerless

The fathers reported having difficulties adapting to the diverse fathering role when their children were in foster care. Informal support from their family was enough for them and preferable to demanding formal support from the child protection authorities. The fathers did not want to interfere and did not plan the visits; instead, the children and family members providing informal support assumed responsibility. They were uncertain about their ability as a father to influence the children’s upbringing.

M: And there are probably lots of things that I should get a hold of, but it’s been going on for so long so, at least I’ve said it now. Now the kids can let me know when they want contact with me. Because as I said, it’s all very complicated, this. (Father no. 4)

I: Has there been anyone close to you, who has supported you?

M: Well that would be my girl’s Mum and Dad, her brother and my brother and sister. (Father no. 5)

The fathers’ cognitive limitations made it difficult to adapt to the child’s development, which was evident to the surrounding people. The fathers reported that the people involved in their situation expected them to be more active in their fathering role.

I: Exactly. What kind of support did you have [before the foster care]? Could you describe it?

M: Yes, no, but we did a lot with PYC [Parenting Young Children Program] when we did, with the Child protection treatment center.

I: Precisely.

M: We did again, there was a lot of that I should see her and so on. And that. Before it was sort of, it was like a door each [separate rooms for the child and the father at home], a TV each, like one each.

I: Yes, you sat and each watched your own TV, yes.

M: And our own door each and things like that.

I: Yes, exactly, now they thought you should do more together [with your daughter], right?

M: Yes.

I: Talk to each other more.

M: Yes. (Father no. 2)

The fathers were uncertain about the future and their fathering role. The visits became less frequent, weakening a fragile relationship with the child. They reported worrying about the void that might be left in the lives of their growing children.

### I am troubled

In this subcategory the fathers revealed the extraordinary complexity of their situation. They understood that their ability was limited. They needed to fill in forms for the social security services to obtain the necessary allowances. They found the paperwork difficult and quickly gave up.

You can get up to ten days or something if you apply specially and I can’t manage an application like that because I don’t know how to deal with the Social Insurance Office. They nearly had me finishing myself off… So I was off work, on sick leave, after separating so I was on my own with the kids so I tried to go for early retirement… But now since he’s been away I’ve been working full time. So the economics of taking care of [my child], the care allowance… it’s a crazy amount. (Father no. 7)

The fathers had employment or other daily occupations they highly valued and did not like to miss. The everyday occupation was considered essential. They worked hard and avoided taking time off, which complicated planning and could affect whether they attended the visitations. One possible consequence was that wanting to fulfill their working role could constitute a barrier to maintaining the father-child relationship and the fathering role.

*I: Yes, right. And what was it that stopped it [the* visits*] from happening? Was it hard to schedule a meeting?*

T: Because of my job, I work so much. It was difficult to take time off.

I: Yes, so that was a problem.

T: Thanks to there being a lot of people working but not many people with a license to drive.

I: Right, OK, yes. So you, you had a hard time getting time off from work then?

T: Yes, especially when you have to do the driving. And take people along. (Father no. 1)

The father’s geographical distance from the child could also make visits difficult. The frequency of visits was also affected when the journeys were expensive.

P: it’s the economics too, it costs quite a lot to get down there, right?

I: Yes, it costs. But if you had the money you would be able to meet him more often?

P: Yes, absolutely. (Father no. 8)

### I am disappointed with the system

In this subcategory the fathers reported being disappointed with several issues in their present life. They were not receiving the support they expected from the system and reported a lack of continuity in contact with the authorities. It seemed that continuity was broken because they often met with new professionals, sometimes due to downsizing or reorganization within the system. In addition, the child protection authorities often made decisions without including the fathers in reflective discussions.

M: … there’s the thing about establishing a contact in the family that gets changed all the time, when what we need is continuity. I mean all these people, our contacts, they get changed all the time. I mean I think he’s on his third social worker or even fourth this time Friday. I say that doesn’t build any confidence. … It doesn’t build my confidence and I honestly just get annoyed. It being like that.

I: Yes, that must be very…

M: You have to start over all the time. (Father no. 7)

The fathers reported having difficulty finding the correct information about their and the children’s rights to see each other, which caused feelings of inadequacy and uncertainty that affected the fathering process. They also revealed that their fathering role was sometimes questioned and not always trusted. This lack of trust made it difficult for the fathers to suggest expanded or more frequent visits, although they desired this.

I: What support have you been offered as a parent in this situation?

M: Actually, nobody’s given me any support.

I: No. No-one?

M: No, not that I’ve got. (Father no. 9)

The fathers experienced that they had it worse than other families. Their needs were complex and extensive, including the foster care child and siblings with disabilities. The need for sustainable support (e.g., structure) was present over the years. Thus, a lack of continuous support could make it difficult for the fathers to keep track of and build trust with the professionals in the system.

I: How would they need to look, the best conditions for things to work properly?

M: The best conditions would be having a day care system that works. So he [the son] can stay at school until I finish my work and that there is somewhere for him to be, a day care centre. It works for other children. So that’s kind of number one that day care works, and then if I can wish for anything it would be going on with the contact with the family worker who comes in maybe twice a month to work on planning and strategy and a bit of conflict management and such. And then a third thing would be if there was some kind of relief family where Benjamin [a pseudonym] likes being. Maybe a couple of months or twice a month or two weekends a month. Something like that, but that’s what we are trying to fix with Grandpa and sort something out, an arrangement or a camp supporting children with LSS support [support according to law FS 1993:387. Act concerning support and service for persons with certain functional impairments]. They get weekend camp once a month and Grandpa one weekend a month. So that’s how we see it, but perhaps a bit as well that we can get a weekend a month where we feel we can cope … (Father no. 7)

The fathers reported how important it was to have employment and that this was why support from the authorities was necessary, specifically when the children were in foster care.

## Discussion

The present study explores the experiences of fathers with neurodevelopmental disabilities who have children in foster care. Specifically, the authors studied their experiences of the fathering role, parent-child visits and formal and informal support. Our analysis revealed two seemingly contradictory categories: *I accept my situation* and *I am frustrated,* suggesting that the fathers were simultaneously content and in submission. This contradiction could also be interpreted as an internal ambiguity of the fathers.

### I accept my situation

All respondents in the current study reported being satisfied with their child’s living arrangement. The overall impression was the presence of trust and confidence, including good cooperation with the foster home and the child protection authorities. Notably, the respondents reported being able to participate as fathers and being part of the parenthood experience. Schofield et al. (2011) also reported parental satisfaction in a more general group of parents with children in foster care. Gordon et al. (2012) specified that this experience might be related to the formal support given to the fathers when support works best. Support may facilitate mutual understanding between fathers and foster families about child-rearing practices. This understanding could benefit the child, given that engaged fathers increase the stability of the situation when children are placed in foster care (Proctor et al., 2011).

The findings of the respondents’ acceptance of the terms and conditions of the foster care arrangement can be explained by the pragmatic strategy of focusing on what works best in daily life. When asked about the fathering role, responses often mirrored the life situation or pointed out what works well. Baum and Negbi (2013) showed that grief seemed to follow a process towards adaption, increased ability to cope with the loss and a matured parental role over time. The respondents may accept the court order due to a similar process. Schofield et al. (2011) reported how parents managed their threatened identity when children were placed in foster care, one strategy being to focus more on work and less on parenting (Ibid.). In the present study all respondents but one had some daily occupation, some in sheltered or supported employment, subsidized to different degrees. The respondents valued having daily work, which contributed to the experience of accepting the situation. Being the financial provider is a defining role in fathering (More and Tarleton, 2022). Symonds et al. (2021), who studied fathers with intellectual disability, emphasized that their descriptions of fatherhood involved at least some childcare and included playing with the child. These observations were also seen in the present study. A previous interview study found that mothers reluctantly accepted that their children were in foster care (Janeslätt et al., 2019). Unlike fathers, the mothers primarily focused on the child rather than work.

### I am frustrated

The respondents in the present study were frustrated and disappointed with the system the professional workers and the child protection authorities represented. According to them, the professionals were incompetent and lacked continuity in their decision-making. They reported feeling uncertain and powerless when the professionals expected them to behave in a certain way. The fathers experienced not being involved, not being included and sometimes not being trusted, confirming the findings of More and Tarleton (2022) and Nygren et al. (2019). In social work practice these authors found that fathers were marginalized and excluded from parenting decisions. According to Nygren et al. (2019), social workers’ reason for exclusion was that the fathers posed a risk of violence, although there was no evidence or a history of violence to support such an accusation.

The respondents also expressed feelings of not being trusted, which could contribute to their general frustration and distress. Despite feeling exasperated and disappointed, the respondents did not object to having their child in out-of-home care. It seems the fathers in the current study feared revealing their vulnerability or grief, a condition Baum and Negbi (2013) also found. Pytlowana and Kroese 2021 and Schofield et al. 2011 showed that parents perceive feelings of blame and shame for releasing their children to foster care. The respondents’ uncertainty and powerlessness could be interpreted as suppressed anger, now expressed in frustration or disappointment. Another reason could be attributed to having ambitions they cannot fulfill. There are indications that they are aware of their shortcomings due to their cognitive limitations, attributed to their experiencing a lack of personal support. One reason for not objecting could be the perceived impossibility of influencing the authorities. Thus, they relinquished care of their child, focusing on achievable goals such as employment.

Parents with neurodevelopmental disabilities from other studies have also experienced the child welfare system as overwhelming (Franklin et al., 2022) and incomprehensible, objecting to being dependent on the system (Janeslätt et al., 2019, Ćwirynkało and Parchomiuk, 2022). These studies suggest that mothers and fathers often experience a lack of support, and the support that is available is not adapted to their individual needs and preferences. Other authors have reported similar findings (e.g., Azar et al., 2013 and Friedrich et al., 2017) A similarity to the mothers in Janeslätt et al. (2019) is the fathers experience of being powerless, frustrated and lacking content in their abilities. Yet, there are differences, mainly related to experiences of the parenting role. Mothers express their grief openly, indicating a need to defend their situation and struggle to show they are responsible adults and mothers (Janeslätt et al., 2019). These characteristics were not found in the fathers of the current study; instead, the fathers expressed feelings of uncertainty and powerlessness.

### Experiences of support

The findings in the present study support the importance of identifying and providing services adapted for fathers with cognitive limitations. These findings are in line with what Azar et al. (2013), Friedrich et al. (2017) and Ćwirynkało and Parchomiuk (2022). These investigators propose that parents with neurodevelopmental disabilities receive services adapted to their cognitive limitations. It is known that support can increase paternal involvement with children. Because the social role of men does not support being a father at a distance, it is imperative to help fathers maintain the relationship with the child and develop ways to be emotionally available to the child in foster care (Grief et al., 2007). The support should be adapted to the cognitive and emotional functions of the individual father (Azar et al., 2013; Symonds et al., 2021; Friedrich et al., 2017). Fathers and mothers may benefit from group interventions that allow them to grow and develop their skills in the reconciliation process (Adolfsson et al., 2021). Offering adapted group support may benefit vulnerable parents and their children, helping to secure their rights and meet the unique needs of the child and family.

### Fathers being simultaneously self-confident but resigned

Living with the circumstances of being a father with neurodevelopmental disabilities and cognitive limitations and children placed in foster care inevitably leads to vulnerability. The difficulties in accepting and managing their life situation, being self-confident and frustrated characterized the fathering role in the current study. On the one hand, the contradiction seemed to be related to the substantial support offered in the child’s upbringing and other needs being met; on the other, the fathers experienced their shortcomings due to their cognitive limitations and being excluded from child-rearing and parenting, with little or no support. Disappointment with the authorities undermines trust and confidence. The view of the social workers of the fathers’ passive behavior could be interpreted in two ways: 1) the fathers accept the situation, which was partly true, or 2) it could be misinterpreted as a lack of interest in the children. This misinterpretation could entail the risk of overlooking the fathers’ need for motivation and adapted support to overcome their sense of hopelessness and powerlessness. This condition and the need for social services to implement adapted services have been discussed elsewhere (Azar et al., 2013).

Another understanding of the two contrasting categories--I accept my situation and I am frustrated--may be related to gender issues, specifically norms of masculinity. Wilson et al. (2012) reasoned that even men with intellectual disability are affected by gendered settings. Yet, there is a dilemma between notions of masculinity and intellectual disability in these men. Having an intellectual disability is often linked to powerlessness and dependence, in contrast to the traits linked to norms of masculinity. This perspective can shed light on the dilemma between the dependency, disadvantage and marginalization related to cognitive limitations and understandings of masculinity connected to strength, independence and power. Moreover, Gordon et al. (2012) outlined the challenges social services face to manage the balancing act between highlighting strengths and identifying needs. The latter is essential if fathers stay engaged and accept the assistance of the welfare system. In the present study fathers reported being involved and independent, prioritizing their daily occupations and accepting what is being provided in support and father-child visits. The social workers should be aware of and find ways to reduce the risk of detachment, supporting an active fathering role striving for engaged and informed fathers. Still, in this challenging work it is essential to find ways to reduce the risk of detachment, supporting an active fathering role and striving for engaged and informed fathers.

### Limitations

This study has some limitations. One weakness is the small sample size. Because recruitment proved challenging, we were forced to rely on other people to recruit respondents. Nevertheless, professionals and the mothers recruiting fathers were aware of the inclusion criteria and used the easy-to-read leaflet. The participants, though few, represent a wide variety of diagnoses and cognitive limitations. One consequence of the small number of respondents is that it does not ensure they represent a variety of ages and life situations to represent the target group fairly.

Because the respondents had cognitive limitations, the interviewers needed to ensure that the questions were understood or reworded when required. Some respondents were talkative during the interviews, whereas others were more reserved. The researchers conducting the interviews are experienced in adapting the interview material and subsequent data analysis to this target group. In the analytical process we sought to analyze the breadth and highlight the commonality of the narratives while still being sensitive to capturing the ambivalence within and between the fathers. Gender and masculinity issues were not included in the interview guide. Nonetheless, in interpreting the findings, these perspectives were useful in understanding the contradictory themes and thus were included in the discussion.

The trustworthiness of this qualitative study was confirmed by establishing credibility, transferability, dependability and confirmability (Nowell et al., 2017). Credibility was addressed through a questionnaire used earlier in a study with parents with neurodevelopmental disabilities and cognitive limitations (Janeslätt et al., 2019). In addition, the respondents were recruited by experienced and skillful professionals and relatives, ensuring they represented the target group of interest. Transferability was determined by presenting the respondents’ demographics as listed in Table 1. Dependability was assured through the systematic analysis, as shown in Table 2. To confirm the results the present study was discussed together with knowledgeable professionals. Finally, the four authors ensured confirmability through thoughtful, ongoing discussions.

### Conclusions

The fathers expressed ambivalence towards acceptance and frustration of the fathering role. Still, their content was evident. The study shows that more needs to be done for these vulnerable fathers with neurodevelopmental disabilities, cognitive limitations and children in foster care, although they seldom request such support. Increased efforts from the social workers may reduce the risk of father resignation because engaged and informed fathers are in the children’s best interests.

## Supplemental Material

Supplemental Material - A father nevertheless: Self-confident but resigned fathers with children in foster care Supplemental Material for A father nevertheless: Self-confident but resigned fathers with children in foster care by Päivi Adolfsson, Helena Lindstedt, Gunnel Janeslätt, Karin Jöreskog in Journal of Intellectual Disabilities
