# Supplementary material for: A father nevertheless: Self-confident but resigned fathers with children in foster care
Source: J Intellect Disabil. 2023 Dec 29;29(2):271–88. doi: 10.1177/17446295231225525 (PMC12084658; doi:10.1177/17446295231225525)
Supplement: Supplemental Material - A father nevertheless: Self-confident but resigned fathers with children in foster care [file sj-pdf-1-jld-10.1177_17446295231225525.pdf]

Appendix

Interview guide

Demographic questions

1. I am:

☐ woman

☐ man

Age: ………………..

2. Housing

☐ An apartment ☐ house other: ……………………………..

3. Civil state

☐ Single ☐ Live together with someone

4. Children:

☐ no

☐ living at home, number: Age:

☐ in foster care, number: Age:

☐ earlier in foster care, number: Age

5. Level of education:

☐ mainstream school ☐ special school ☐ collage

6. employment status:

☐ do not work ☐ Yes with what? ……………………………………..

7. Do you have a disability or diagnoses that influences your everyday life?

☐ no ☐ yes what? ……………………………………..

Interview questions

How do you experience your parenting role today?

How are the visits with your children in foster care?

Can you decide what you to do during the visits?

Can you choose when, where and how long you will be together with you child?

Can you influence the child's everyday life in the foster home?

How do you experience you relation to the foster parents is today?

Do they treat you as the father of your child; do they support you in your fathering role?

Can you influence the foster home and your child's everyday life there?

Do you need support in your parenting role get any support?

What support have you been offered as a parent in this situation? Who has offered that? Has that support been adapted to your cognitive disability?

Is it right type of support?

Do you get enough support for your needs

Thank you for your participation!
